# Supplementary material for: Role of Diacylglycerol Kinases in Acute Myeloid Leukemia
Source: Biomedicines. 2023 Jul 1;11(7):1877. doi: 10.3390/biomedicines11071877 (PMC10377028; doi:10.3390/biomedicines11071877)
Supplement: Supplementary file 1 [file biomedicines-11-01877-s001.zip › biomedicines-2379974-supplementary.pdf]

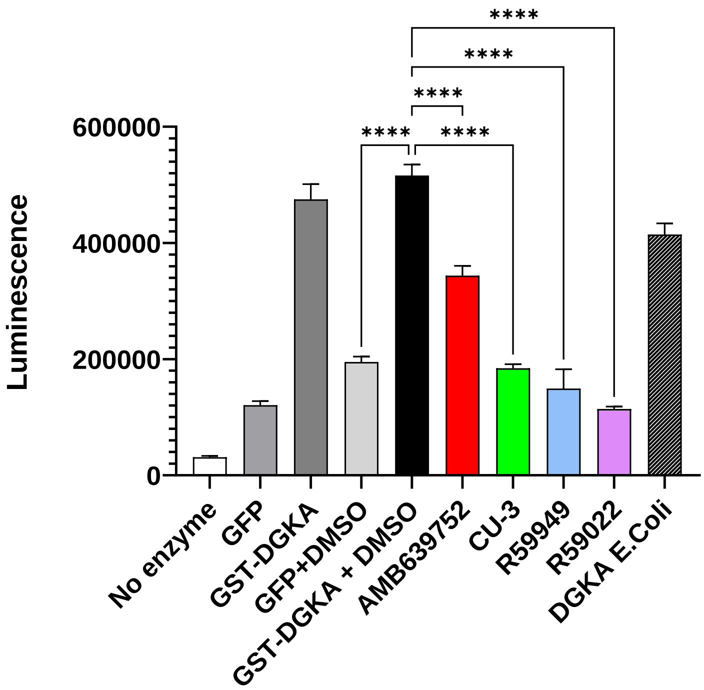

Supplementary Figure S1 Effect of DGK inhibitors on DGKA enzymatic activity

The activity of in-house purified GST-DGKA was measured using the kinase enzyme system with the ADP-Glo^{TM} assay (Promega) in the presence or absence of different DGK inhibitors at a final concentration of 400 µM (R59949 IC_{50} 267 µM). Data are shown as the mean ± SEM and the experiment was performed in quadruplicate. Asterisks *, **, *** and **** denote a p-value significance of ≤ 0.05, ≤ 0.01, ≤ 0.001 and ≤ 0.0001 respectively.

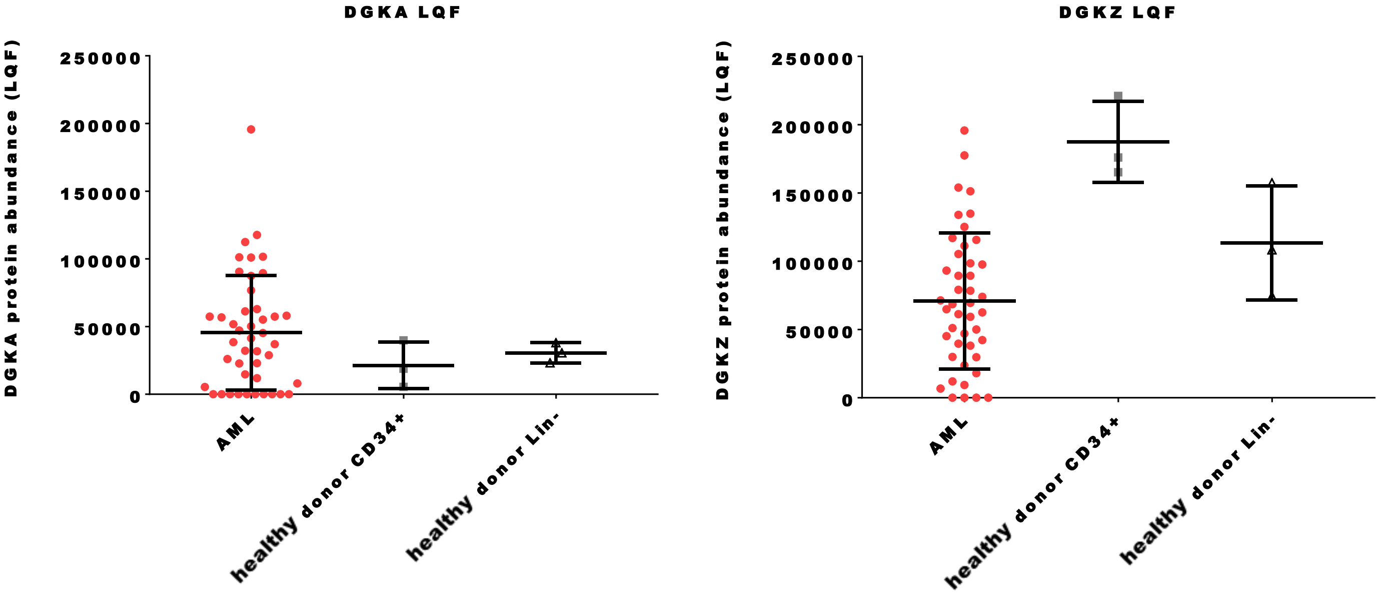

Supplementary Figure 2 DGKA and DGKZ protein expression in AML

Label-free-quantification (LFQ) protein abundance measurements from 44 adult patients from the TCGA dataset with de novo AML as well as three healthy donors [30]. Due to reduced number of controls no statistics were performed.

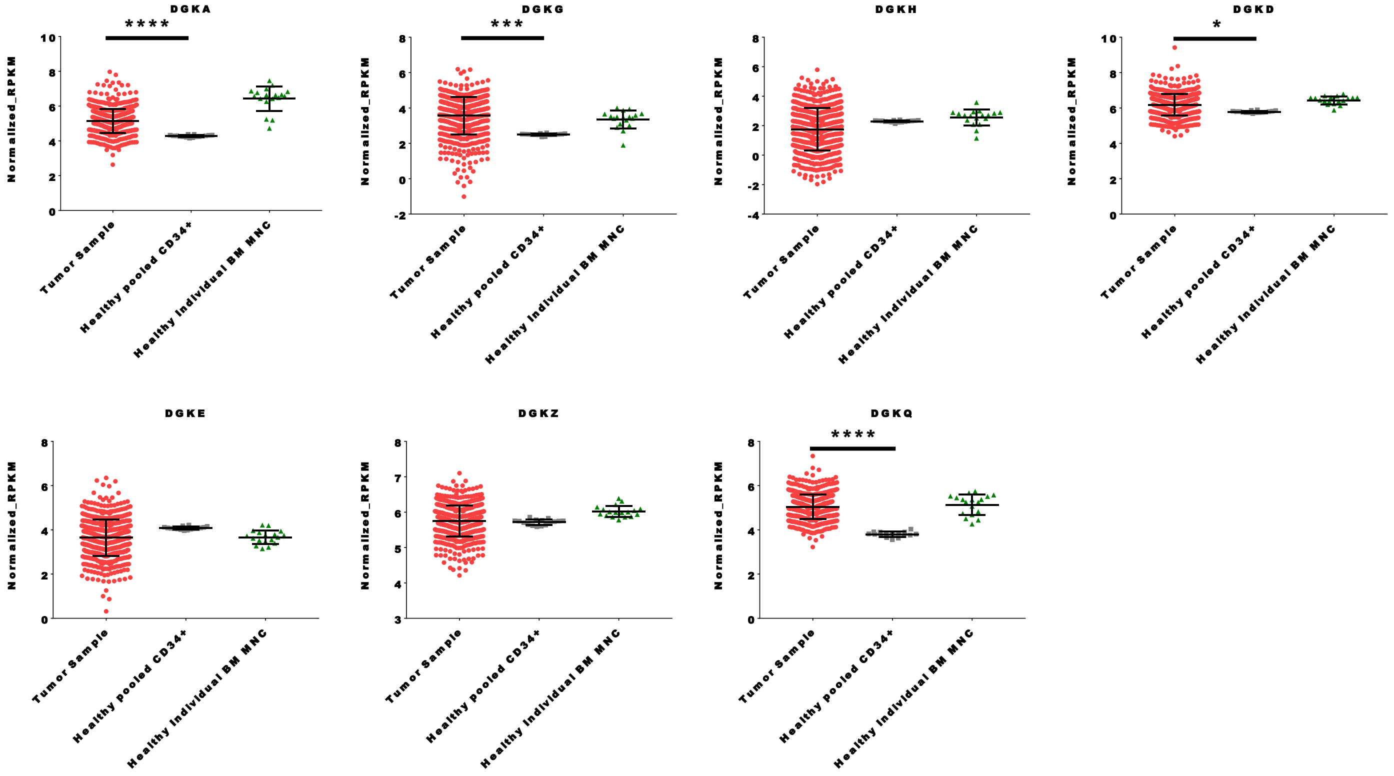

Supplementary Figure 3 DGK expression in AML

Data from BeatAML database [26] comprising 671 tumor, 16 healthy pooled CD34+ and 18 healthy individual BM MNC samples, are shown as Normalized_RPKM, mean ± SD. Tumor sample and healthy pooled CD34+ cells are compared using one-way ANOVA, p<0.05 *, <0.01 **, 0.001***, 0.0001 ****. RPKM: Reads Per Kilobase per Million.

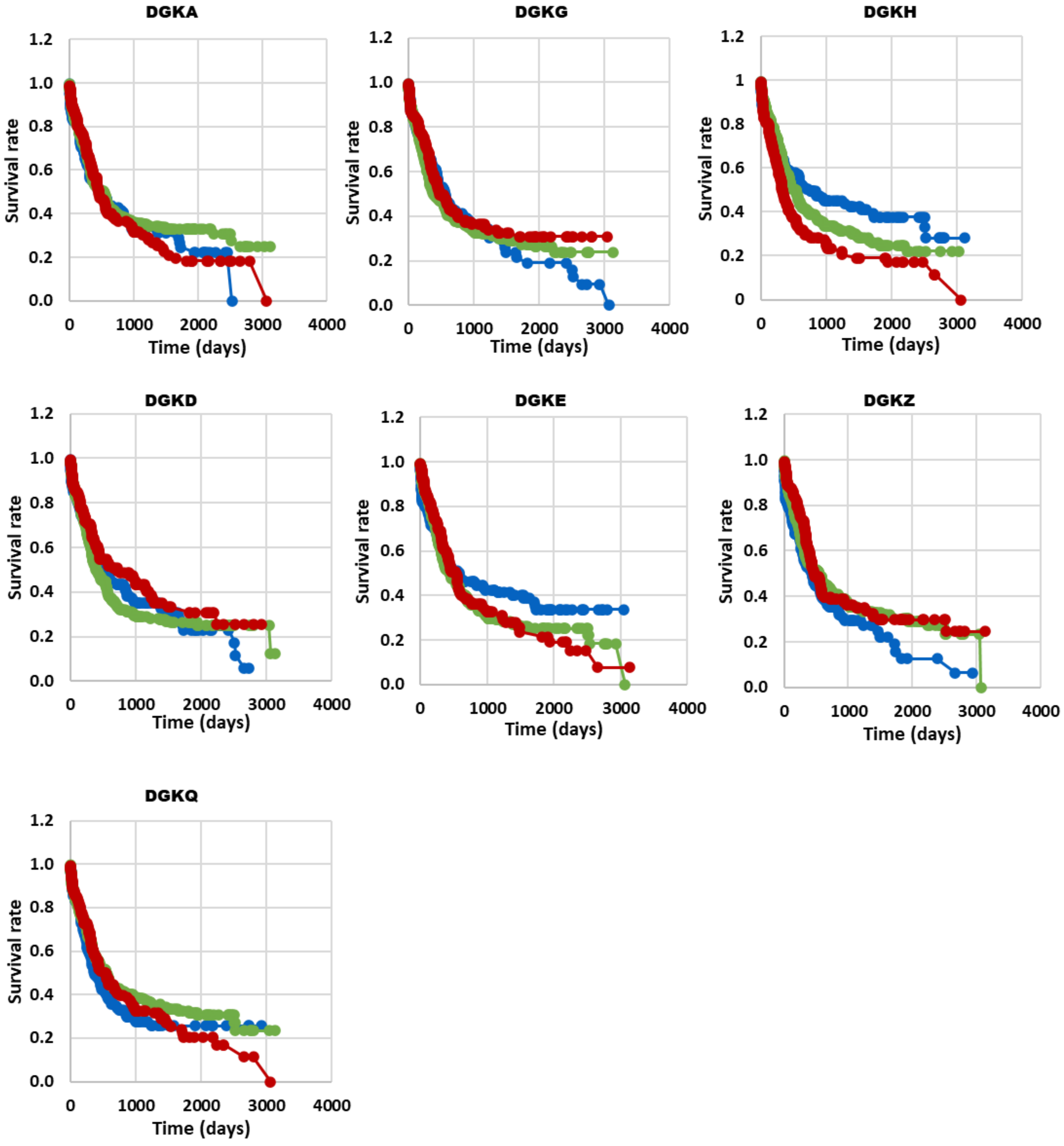

Supplementary Figure 4 DGK expression and survival correlation in AML (BeatAML database)
High DGK group is shown in **red**, low DGK in **blue**, normal levels in **green**.

- *DGKA* n(up)=142, n(no change)=260, n(down)=136 with p=0.52
- *DGKG* n(up)=139, n(no change)=254, n(down)=134 with p=0.54
- *DGKH* n(up)=133, n(no change)=261, n(down)=142 with p<0.01 *
- *DGKD* n(up)=139, n(no change)=251, n(down)=141 with p=0.18
- *DGKE* n(up)=136, n(no change)=259, n(down)=136 with p=0.29
- *DGKZ* n(up)=140, n(no change)=261, n(down)=136 with p=0.08
- *DGKQ* n(up)=134, n(no change)=266, n(down)=140 with p=0.22

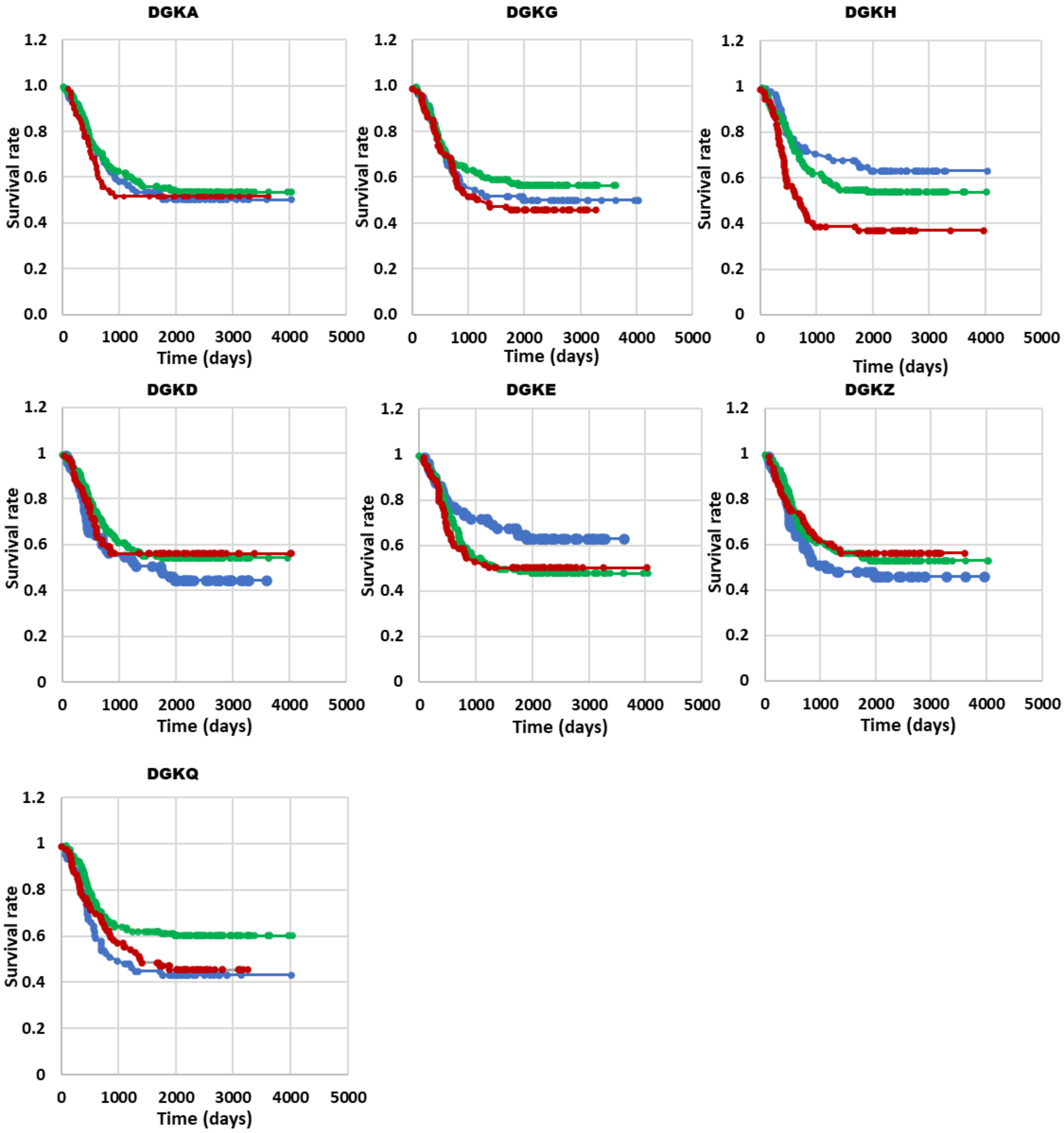

Supplementary Figure 5 DGK expression and survival correlation in AML (TARGET database)
High DGK group is shown in **red**, low DGK in **blue**, normal levels in **green**.

- *DGKA* n(up)=77, n(no change)=152, n(down)=72, with p=0.72
- *DGKG* n(up)=72, n(no change)=154, n(down)=79 with p=0.37
- *DGKH* n(up)=69, n(no change)=151, n(down)=80 with p<0.01 *
- *DGKD* n(up)=77, n(no change)=148, n(down)=75 with p=0.33
- *DGKE* n(up)=76, n(no change)=148, n(down)=78 with p=0.09
- *DGKZ* n(up)=77, n(no change)=147, n(down)=74 with p=0.40
- *DGKQ* n(up)=79, n(no change)=145, n(down)=78 with p=0.02 *

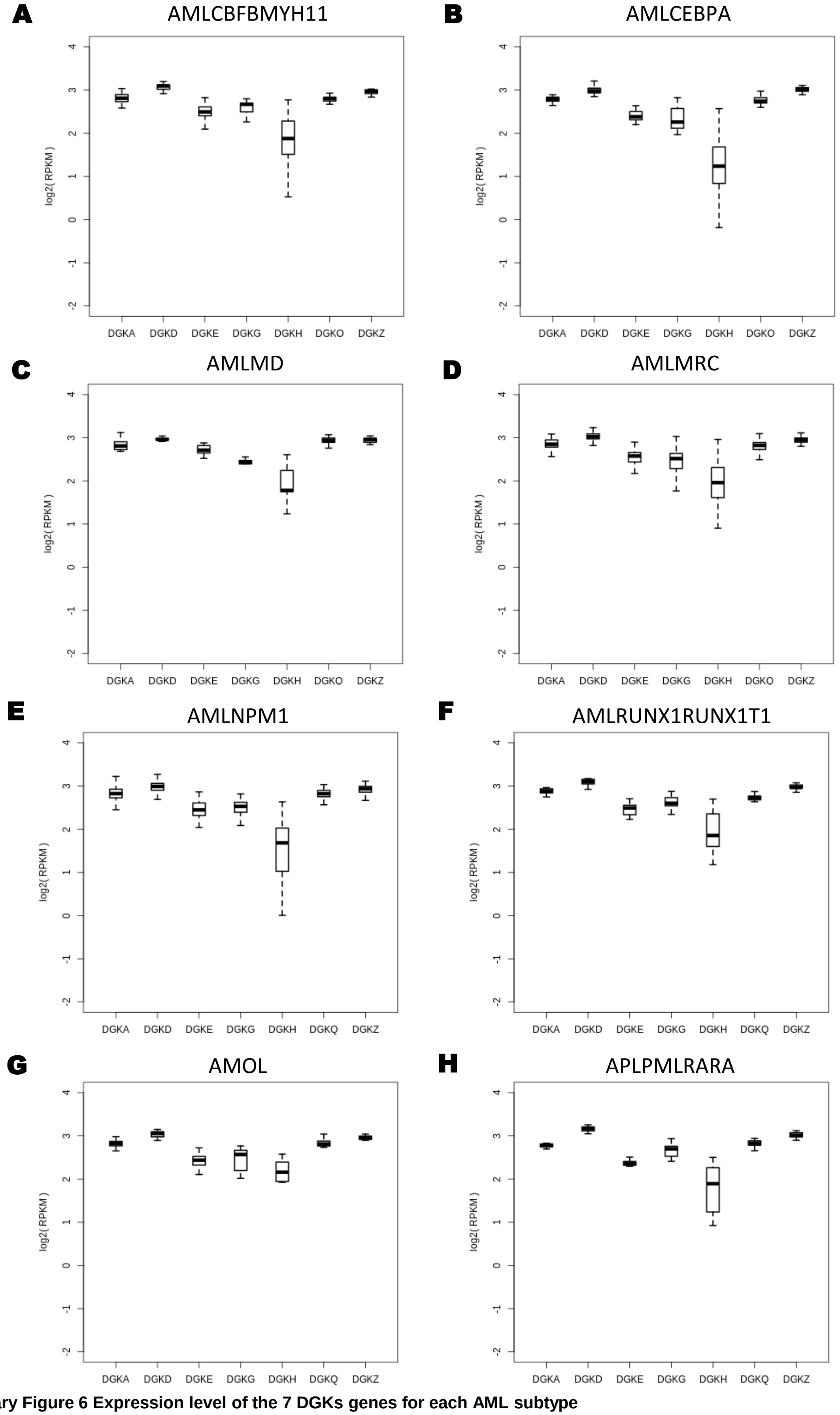

Supplementary Figure 6 Expression level of the 7 DGKs genes for each AML subtype

Based on RNA-seq data obtained from cBioPortal (OHSU, Nature 2018 dataset), expression values are in log_2(RPKM). Boxplots are labelled by the Oncotree Code.

A. Acute myeloid leukemia with CBFB::MYH11 fusion
B. Acute myeloid leukemia with CEBPA mutation
C. Acute myeloid leukemia with minimal differentiation
D. Acute myeloid leukemia, myelodysplasia-related
E. Acute myeloid leukemia with NPM1 mutation
F. Acute myeloid leukemia with RUNX1::RUNX1T1 fusion
G. Acute monocytic leukemia
H. Acute promyelocytic leukemia with PML::RARA fusion

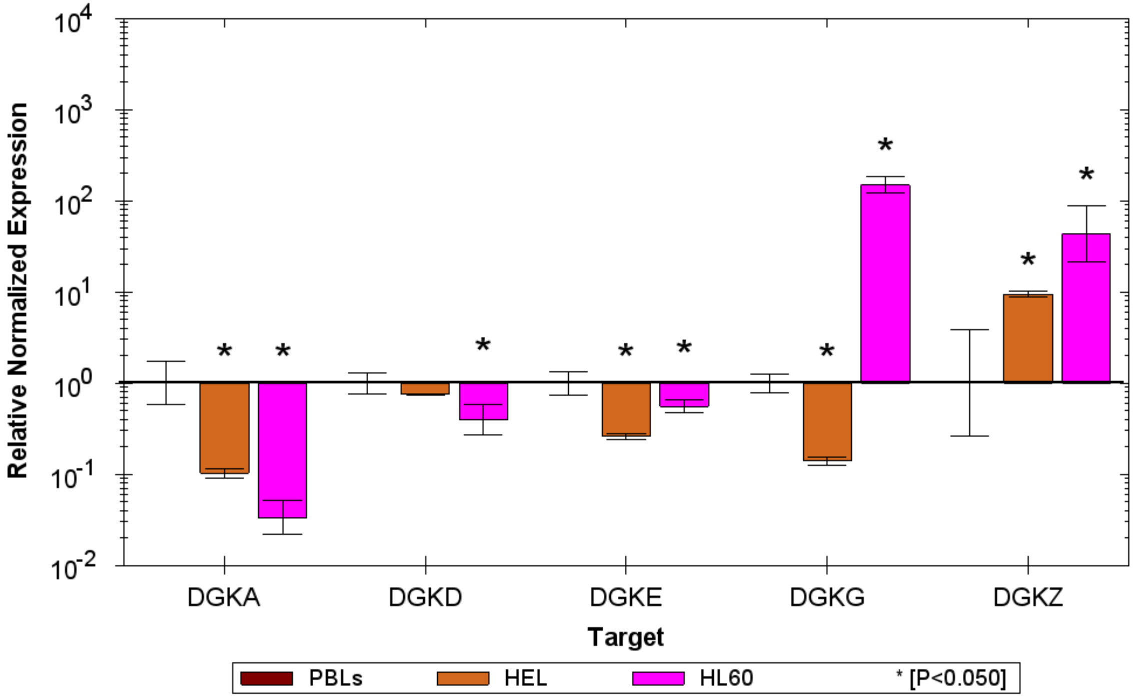

Supplementary Figure 7 Relative gene expression of DGKs main isoforms.
Isoform expression was measured in the real-time polymerase chain reaction, in HEL and HL60 cell lines compared to PBLs. * = p<0.05.

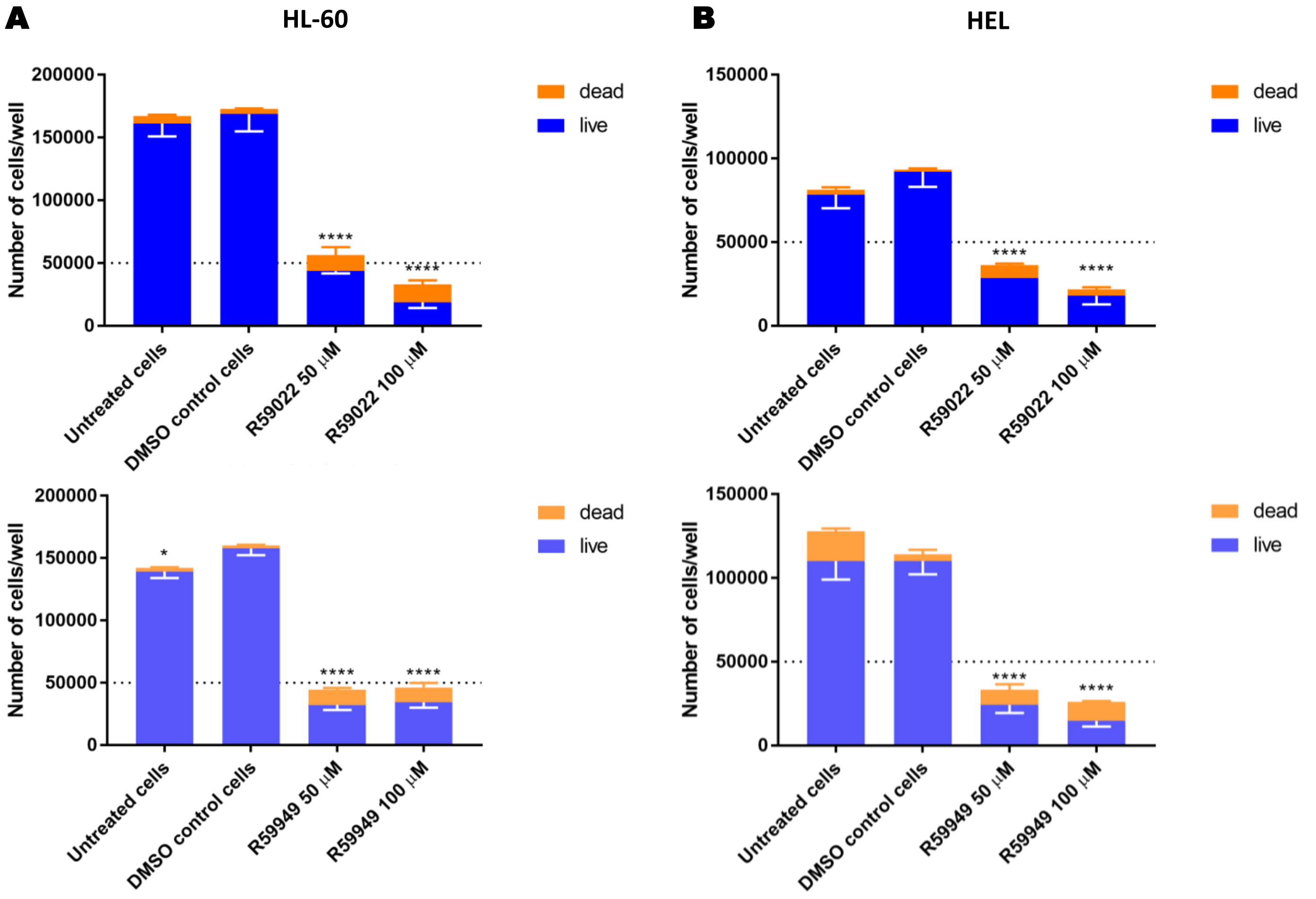

Supplementary Figure 8 Trypan blue live/dead cell count

Representative experiment of Trypan blue live/dead cell count to assess the effect of R59022 and R59949 on cancer cells, namely HL-60 (panel A) and HEL (panel B), after 48h incubation in the indicated conditions. Experiments was run in triplicate, graphed as number of live/dead cells ± SD and analyzed with GraphPad Prism 7 (ordinary one-way ANOVA), p<0.0001 ****. The same trend was observed in three independent experiments performed in the same conditions.
